# Parturition Signaling by Visual Cues in Female Marmosets (*Callithrix jacchus*)

**DOI:** 10.1371/journal.pone.0129319

**Published:** 2015-06-05

**Authors:** Laís Alves Antonio Moreira, Danilo Gustavo Rodrigues de Oliveira, Maria Bernardete Cordeiro de Sousa, Daniel Marques Almeida Pessoa

**Affiliations:** 1 Laboratory of Sensory Ecology, Department of Physiology, Federal University of Rio Grande do Norte, Natal, RN, Brazil; 2 Laboratory of Behavioral Endocrinology, Department of Physiology, Federal University of Rio Grande do Norte, Natal, RN, Brazil; 3 Laboratory for the Advanced Study of Primates, Department of Physiology, Federal University of Rio Grande do Norte, Natal, RN, Brazil; 4 Laboratory of Neuroscience and Behavior, Department of Physiological Sciences, University of Brasília, Brasília, DF, Brazil; 5 Brain Institute, Federal University of Rio Grande do Norte, Natal, RN, Brazil; University College London, UNITED KINGDOM

## Abstract

New World monkeys have polymorphic color vision, in which all males and some females are dichromats, while most females are trichromats. There is little consensus about which selective pressures fashioned primate color vision, although detection of food, mates and predators has been hypothesized. Behavioral evidence shows that males from different species of Neotropical primates seem to perceive the timing of female conception and gestation, although, no signals fulfilling this function have been identified. Therefore, we used visual models to test the hypothesis that female marmosets show chromatic and/or achromatic cues that may indicate the time of parturition for male and female conspecifics. By recording the reflectance spectra of female marmosets’ (*Callithrix jacchus*) sexual skin, and running chromatic and achromatic discrimination models, we found that both variables fluctuate during the weeks that precede and succeed parturition, forming “U” and inverted “U” patterns for chromatic and achromatic contrast, respectively. We suggest that variation in skin chroma and luminance might be used by female helpers and dominant females to identify the timing of birth, while achromatic variations may be used as clues by potential fathers to identify pregnancy stage in females and prepare for paternal burdens as well as to detect oestrus in the early post-partum period.

## Introduction

As social animals, primates use different sensory modalities (e.g. acoustic, chemical, tactile and visual) to convey information about social and sexual status to conspecifics [1, 2]. Among these modalities, visual signals are widely used, especially color signals, since primates are the mammalian group that displays the greatest variety of colors in their skin and fur [3] and carries the best color vision [4]. Yet, color vision is highly variable among primates [5]. While all Old World monkeys have uniform trichromatism, New World monkeys exhibit a color vision polymorphism that is controlled by a single polymorphic gene locus on the X-chromosome, resulting in dichromatism (roughly equivalent to human red-green color blindness) in males and homozygous females and trichromatism (roughly equivalent to humans with normal color vision) in heterozygous females [6]. Four major hypotheses have been tested regarding the evolution of primate color vision [7]: detection of food (e.g. ripe fruits, young leaves and insects) [8–12], detection of predators [13], social dynamics [14] and sexual selection [15]; although long-term fitness data suggest that balancing selection maintains color vision variation and that no phenotype is superior [9]. Hypotheses relating sexual selection and color vision have not been studied in New World primates yet, even though Old World primates [15–18] and Strepsirhines [19] have already been considered. This gap is unfortunate, since 31% of primate species inhabit the New World [20].

Even if studies on chromatic signaling are still lacking, research in reproductive behavior and physiology suggests that marmoset and tamarin males are able to perceive the timing of conception and pregnancy stages in females [21–24]. In wild and captive marmosets (*Callithrix jacchus*), reproductive pairs display higher rates of contact and mate-guarding behavior during conception [21, 22]. In wild lion tamarin (*Leontopithecus rosalia*) groups, males direct aggressions toward immigrant females when resident females are pregnant [23]. Experiments in captive *Callithrix jacchus* and *Saguinus oedipus* show that breeding males go through hormonal and physical changes and gain weight when females are pregnant [24]. Apparently, males identify conception and pregnancy stage in order to enhance their food intake or change their metabolism so that the future fathers are prepared for their paternal duties [24], such as carrying offspring [25]. In this species the number of adult males in the group is related to infant survival [26] and two or more non-reproductive individuals in most free ranging groups help in the infant carrying [25]. Interestingly, male behavioral and physical changes occur before fetal growth and female weight gain [24], indicating the existence of sensory cues other than a swollen abdomen. This suggests that the detection of socio-reproductive signals by males is highly adaptive, especially in socially monogamous species with extensive paternal care investment, such as marmosets [25], where the energetic cost of reproduction is very high, exemplified by the high litter-maternal weight ratio at birth [27], as well as the occurrence of lactation and ovulation during the early post-partum period [28].

Common marmosets (*Callithrix jacchus*) are cooperative breeders and, usually, only the dominant female reproduces in the social group whereas ovulation of subordinates is suppressed. The mechanisms underlying the reproductive failure of subordinates were hypothesized into two scopes: 1) temporal, which means that reproduction in subordinates could be suppressed prior (pre) or after (post) conception; 2) mechanistic, which might involve physiological and/or behavioral events [29]. Under this perspective, the proximate regulation of this singular breeding in female callitrichid primates seems to be a multifactorial combination of cues that probably arises from dominant females.

We know that olfactory cues are involved in sexual arousal in common marmosets since single adult males increase testosterone levels in the presence of scent secretions from ovulatory females [30]. So, it is possible that olfactory cues are also implicated in the physiological suppression of ovulation in subordinate females. However, the flexibility of responses observed on the physiological profiles of subordinates, depending on the context [31, 32], suggests that other sensory cues could also be involved. For instance, as also described for scent transfer, simple visual exposure to dominant females has been shown to delay ovulation in subordinates [33]. In free-ranging conditions, in contrast to olfactory information, visual cues are infinitely faster, extremely directional and, depending on the phytophysiognomy (e.g. forest, woodland, grassland) and/or location within the vegetation (e.g. forest edge, forest canopy, understory), might be transmitted at longer or shorter distances between signalers and receivers [34], playing a different role in primate communication. In addition, vision is the most developed sense in primates, showing more than 30 different brain areas specialized in visual processing [35], and considering the importance of studying the relative contribution of the different sensory modalities to animal communication [36, 37], visual cues should be regarded, at least, as a complementary indication of the physiological state of females.

Among mammals, primates exhibit striking examples of skin and pelage color variation [2, 3]. The role of hormones in modifying hue, luminance, size and texture of external genitalia is well documented for Old World primates [38]. However, only one study has been conducted in New World primates, and considered the course of pregnancy in marmosets [39]. In this study, a color change in the vulvar mucosa was observed during the last four weeks of gestation [39], which might be a means of predicting imminent parturition. This was the first evidence of skin color changing in a New World monkey, but it considered human vision. Therefore, since visual models that consider the visual system of the viewer may provide more accurate information on the form and function of visual signals than human subjective perception [40, 41], we used chromatic and achromatic discrimination models to test the hypothesis that female marmosets show hue and/or luminance cues that may indicate the time of parturition for male and female conspecifics.

## Material and Methods

### Study animals

Following the three Rs (Replacement, Reduction and Refinement) for more ethical use of animals in testing [42], four pregnant female common marmosets (*Callithrix jacchus*), aged 4.75 ± 1.5 years and with previous experience in maternity, were housed with their reproductive partners and offspring in outdoor enclosures (1.0 x 2.0 x 2.0 m—for more details see [43]), under natural conditions of temperature, humidity and illumination, at the Laboratory for the Advanced Study of Primates, a marmoset colony of the Federal University of Rio Grande do Norte (UFRN), Brazil. The lateral walls (2.0 x 2.0 m), separating neighboring families, as well as the enclosures’ floor (1.0 x 2.0 m), were made of brick and cement. Ceiling, front and rear walls (1.0 x 2.0 m) were constructed with wire mesh, allowing the subjects to have olfactory, acoustical and visual contact with other families and with the surrounding vegetation. A roof of ceramic tiles, covering three quarters of the enclosures’ ceiling, sheltered the animals and allowed them to have a proper sunbath. Enclosures were enriched with wood perches, concrete platforms, ropes and nest platforms. Water was available ad libitum and food was provided twice a day (7:00–9:00 a.m.; 1:00–3:00 p.m.). Food consisted of seasonal fruits and a protein rich preparation that supplemented the diet.

Gestational age was estimated by transabdominal palpation [39] and counting backward from the day of parturition. All animals were in excellent health and were previously habituated to researcher manipulation in order to reduce the stress of capture and containment necessary for data collection. During spectral reflectance measurement, the animals were immobilized by suitable equipment (S1 Fig), to which they had been previously habituated (seven days of habituation before data collection). After immobilization the subjects were rewarded with a sugar rich solution (S1 Fig), which ensured their quick habituation to the procedure without the need of anesthesia. Blood samples were also collected as part of the requirements for an unrelated study. The entire procedure, including color measurements and blood collection, took no longer than five minutes. No animals were sacrificed. The experimental procedures were in accordance with the Guidelines for the Care and Use of Mammals in Neuroscience and Behavioral Research of the National Research Council.

### Data collection

**Fig 1:**
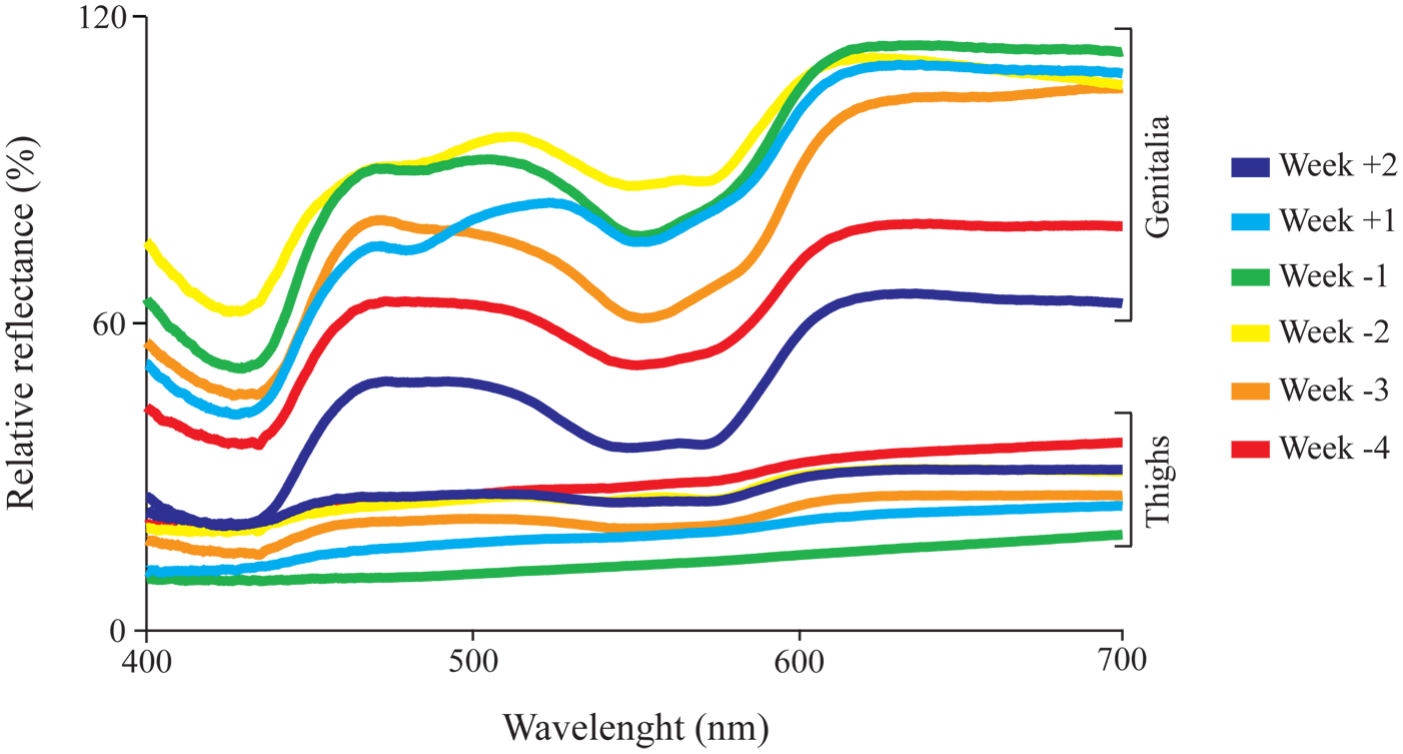
Skin reflectance spectra at different stages of pregnancy. Reflectance spectra from the genitalia and the thighs of one subject (Female C) measured throughout two weeks following (+1 to +2) and four weeks preceding (-4 to -1) parturition.

Data collection was performed once a week, between 9:00 a.m. and 12:00 p.m. A total of 96 reflectance spectra (see Fig 1 for examples of skin spectra), from skin on the right and left side of female genitalia and skin on the right and left inner thighs (S1 Fig), were measured and subsequently averaged (right and left sides), resulting in 48 spectra (24 from genitalia and 24 from thighs—see S1 Dataset for raw values). The choice of thighs as a perigenital region, to be compared against the genitalia, took into consideration which areas would be visualized as surrounding the females’ genitalia during a genital display a few meters away from the viewer. In a previous study, a color change in the vulvar mucosa of female marmosets was observed during the last four weeks of gestation [39]. So, from March to October 2012, measurements were taken every Tuesday, during the last four weeks of pregnancy and first two weeks succeeding parturition. Data that, eventually, had been collected outside this window of six weeks, for only one or two individuals, were disregarded because of their small sample size. The month preceding parturition was divided into weeks, such that parturition day was labeled “0”, the four weeks preceding parturition were labeled “-1” to “-4” and the two weeks following parturition were labeled “+1” and “+2”.

Measurements were always taken immediately after system calibration, at an angle of 45 degrees and 5 mm from the skin, through a USB4000-UV-VIS spectrometer coupled to a fibre-optic probe (R400-7-VIS/NIR), with probe holder (RPH-1), supplied by an LS-1 light source and connected to a notebook running SpectraSuite software (Ocean Optics, Dunedin- FL, USA).

### Data analyses

#### Perceptual analyses

A widely used way of assessing how two patches appear as perceptually different to a receiver is by using the receptor noise model, which estimates the distance between two spectra in the chromatic space in just noticeable difference (JND) units [44]. JND is a perceptual unit in which chromatic contrast can be either perceptible (≥ 1 JND) or not (< 1 JND) [45]. Moreover, the performance of two phenotypes can be considered significantly different when the difference in chromatic contrast from each phenotype exceeds 1 JND [44]. Through this model we evaluated the chromatic contrast between female marmoset genitalia and inner thighs, considering all color vision phenotypes present in the species: three dichromats (with maximum spectral sensitivity of cones at 430/543, 430/556 and 430/562 nm) and three trichromats (with cone peak sensitivities at 430/543/556, 430/543/562 and 430/556/562 nm) [6]. Additionally, we also assessed the achromatic contrast for all phenotypes [41]. All the visual signal analyses were carried out using pavo [46], an R package.

#### Statistical analyses

Two separate linear mixed models (LMM), one for comparing chromatic JND and another for comparing achromatic JND, were applied to our data. Differences in JND ranks between phenotypes and between weeks to parturition were compared. Individuals (females) were included in the models as a random effect, while phenotypes and weeks were included as fixed effects. Fixed effects’ interaction was also considered in the models. Pairwise comparisons, using the least squares means, were conducted through Tukey's test. Confidence levels were always set to 0.95. All statistical analyses were carried out using the following R 3.1.2 packages: nlme, lme4 and lsmeans.

### Ethics Statement

The research protocol was approved by our named Institutional Animal Care and Use Committee (IACUC) or ethics committee, the Animal Research Ethics Committee of the Federal University of Rio Grande do Norte (Comitê de Ética no Uso de Animais da Universidade Federal do Rio Grande do Norte—CEUA/UFRN) (Permit number: 061/2011) and adhered to the legal requirements of Brazilian law.

## Results

**Fig 2:**
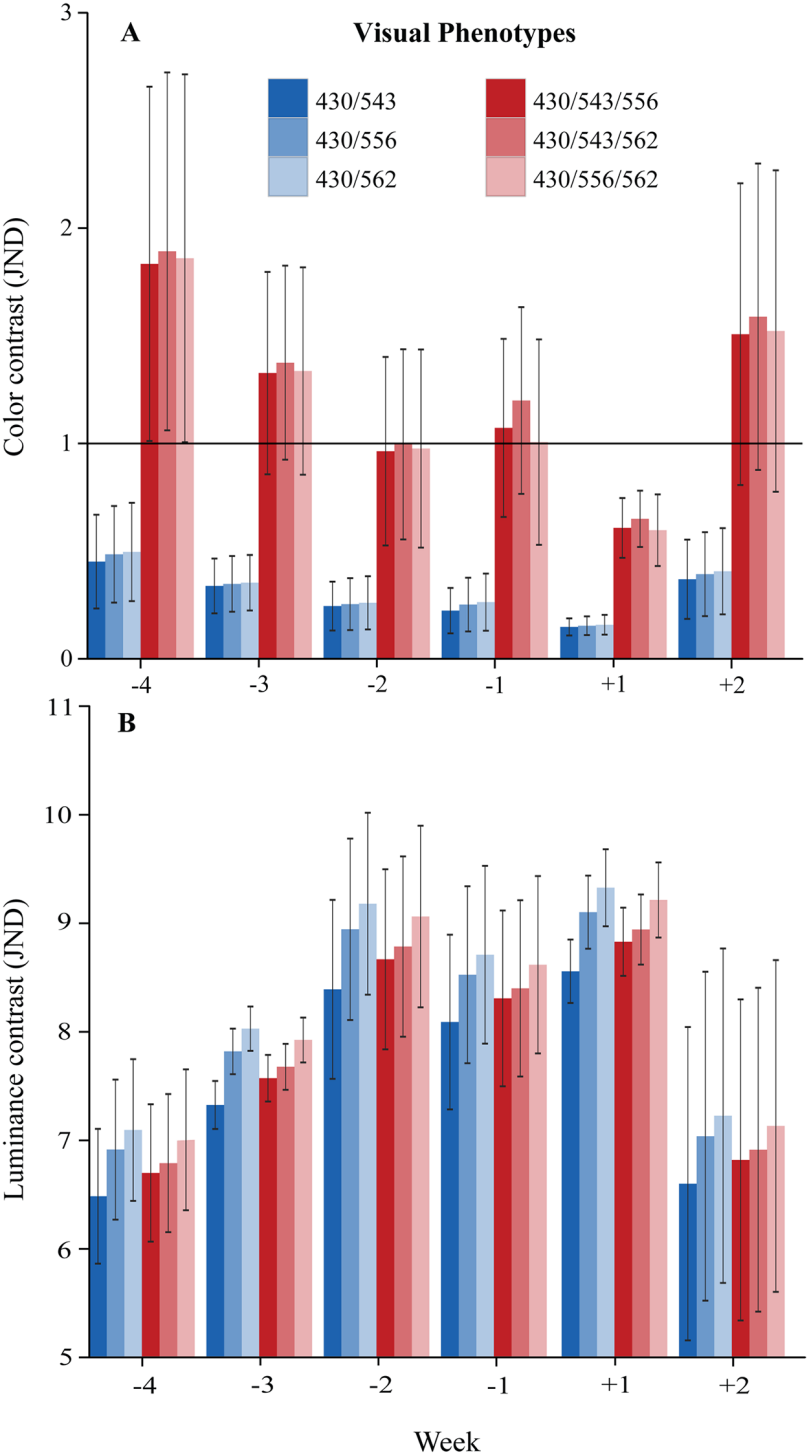
Variation in females’ chromatic and achromatic signals over the weeks. Mean chromatic (A) and achromatic (B) contrast between genitalia and thighs of four pregnant females during four weeks preceding (-4 to -1) and two weeks following (+1 to +2) parturition. Modeled for dichromatic (430/543, 430/556, 430/562) and trichromatic (430/543/556, 430/543/562, 430/556/562) vision. The black horizontal line indicates the perceptual threshold of 1 JND (A). Whiskers represent the standard error of the mean.

**Fig 3:**
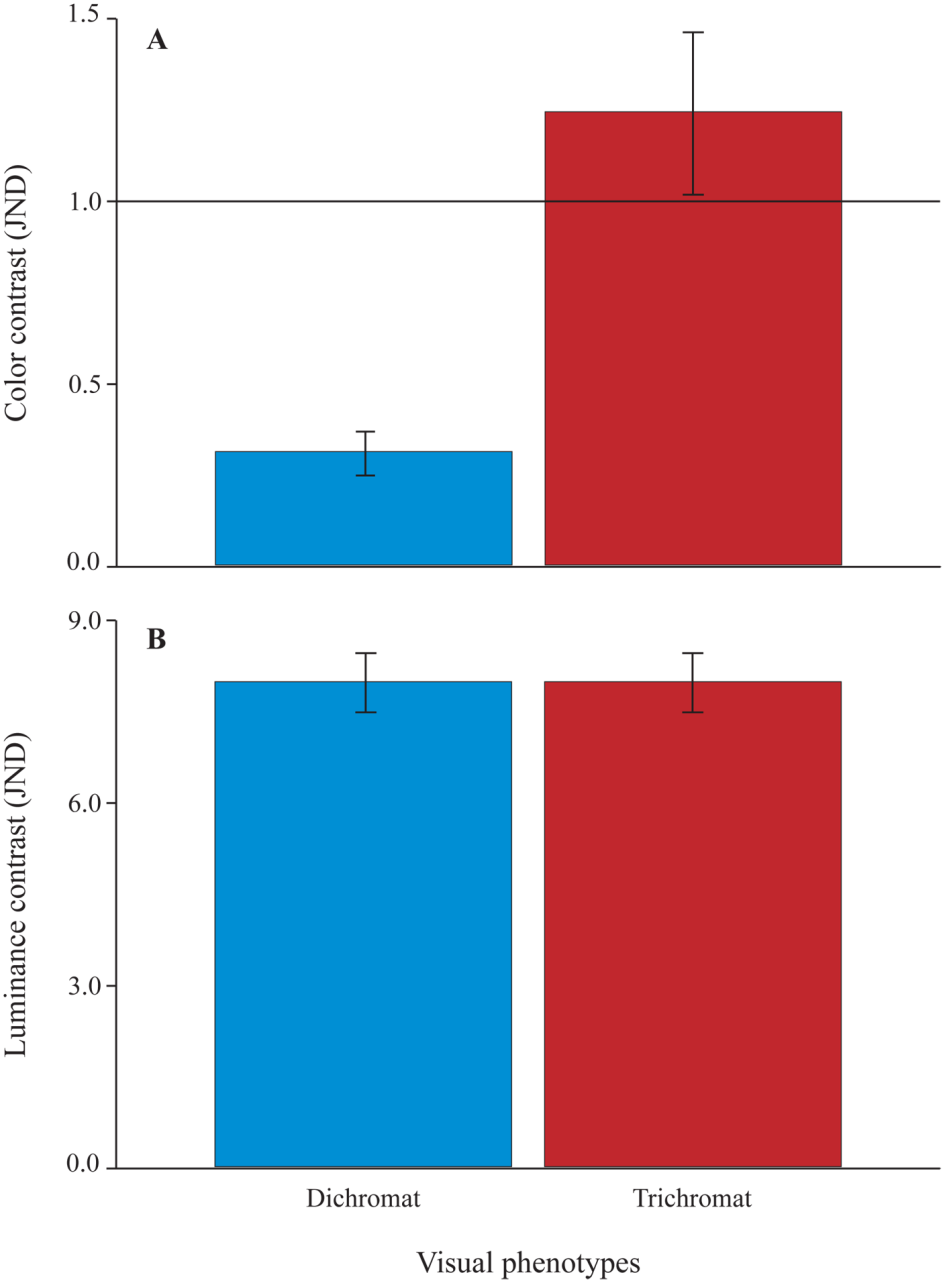
General chromatic and achromatic constrasts for dichromats and trichromats. Mean chromatic (A) and achromatic (B) contrast, between genitalia and thighs of pregnant females, modeled for dichromatic and trichromatic vision. All JND scores (from four females, six weeks and three different phenotypes) presented in Fig 2 have been averaged for each phenotype. The black horizontal line indicates the perceptual threshold of 1 JND (A). Whiskers represent the standard error of the mean.

A reduction in chromatic contrast throughout the last four weeks of pregnancy, followed by an increase in the second week postpartum, forming a “U” feature, was found for all phenotypes (Fig 2A). Chromatic contrast exceeded 1 JND for all trichromats in weeks -4, -3, -1 and +2, while no perceptual chromatic difference between genitalia and thighs were found in week +1. Phenotype 430/543/562 was the only trichromat that should perceive a difference in chroma in week -2 (Fig 2A). By contrast, dichromats never achieved the boundary of 1 JND in any week analyzed (Fig 2A). Statistically, we found that trichromats, when compared to dichromats, reached significantly (*F*_*5*,*105*_ = 14.4253; *p*<0.0001) higher values of chromatic JND (Fig 3A). Results from post-hoc analysis (Table 1) show that dichromatic phenotypes did not differ from one another, but presented significantly lower values of chromatic contrast when compared to any trichromatic phenotype. Trichromats also did not differ from each other. Variation of chromatic contrast over the weeks did not reach significance (*F*_*5*,*105*_ = 1.8678; *p* = 0.1062). No interaction between phenotype and time period was found (*F*_*25*,*105*_ = 0.0755; *p* = 1.0000).

**Table 1:** Output from post-hoc analysis (Tukey's test), showing which phenotypes were significantly different from each other with respect to chromatic contrast.

Comparison	Estimate	SE	df	t.ratio	p.value
D1 vs. D2	-3.0417	9.4327	105	-0.3220	0.9995
D1 vs. D3	-4.7917	9.4327	105	-0.5080	0.9958
**D1 vs. T1**	**-47.9167**	**9.4327**	**105**	**-5.0800**	**<.0001**
**D1 vs. T2**	**-52.7500**	**9.4327**	**105**	**-5.5920**	**<.0001**
**D1 vs. T3**	**-45.0000**	**9.4327**	**105**	**-4.7710**	**0.0001**
D2 vs. D3	-1.7500	9.4327	105	-0.1860	1.0000
**D2 vs. T1**	**-44.8750**	**9.4327**	**105**	**-4.7570**	**0.0001**
**D2 vs. T2**	**-49.7083**	**9.4327**	**105**	**-5.2700**	**<.0001**
**D2 vs. T3**	**-41.9583**	**9.4327**	**105**	**-4.4480**	**0.0003**
**D3 vs. T1**	**-43.1250**	**9.4327**	**105**	**-4.5720**	**0.0002**
**D3 vs. T2**	**-47.9583**	**9.4327**	**105**	**-5.0840**	**<.0001**
**D3 vs. T3**	**-40.2083**	**9.4327**	**105**	**-4.2630**	**0.0006**
T1 vs. T2	-4.8333	9.4327	105	-0.5120	0.9956
T1 vs. T3	2.9167	9.4327	105	0.3090	0.9996
T2 vs. T3	7.7500	9.4327	105	0.8220	0.9629

Bold text has been used to emphasize statistically significant differences.

D1 = phenotype 430/543;

D2 = phenotype 430/556;

D3 = phenotype 430/562;

T1 = phenotype 430/543/556;

T2 = phenotype 430/543/562;

T3 = phenotype 430/556/562.

Achromatic contrast (Fig 2B) showed an inverted “U” pattern of variation compared to chromatic contrast. It was inferred to be always high and perceptible when modeled for dichromats and trichromats throughout all the weeks studied (Fig 2B). The lowest achromatic JND values were found in weeks -4 and +2. These weeks differed perceptually (the difference in achromatic contrast from one week and another exceeded 1 JND) from those weeks around parturition (weeks -2, -1 and +1), which presented the highest achromatic JND scores (Fig 2B). Differently from chromatic contrast, values of achromatic JND did not differ statistically (*F*_*5*,*105*_ = 0.7539; *p* = 0.5851) between dichromats and trichromats (Fig 3B). On the other hand, variation of achromatic contrast over the weeks (Fig 2B) was significant (*F*_*5*,*105*_ = 6.2992; *p*<0.0001). Results from post-hoc analysis (Table 2) show that weeks flanking parturition presented significantly higher values of achromatic contrast when compared to weeks that were farther positioned in time with respect to the birth day. No interaction between phenotype and time period was found (*F*_*25*,*105*_ = 0.0193; *p* = 1.0000).

**Table 2:** Output from post-hoc analysis (Tukey's test), showing which weeks were significantly different from each other with respect to achromatic contrast.

Comparison	Estimate	SE	df	t.ratio	p.value
-4 vs. -3	-12.0000	11.7020	105	-1.0250	0.9084
**-4 vs. -2**	**-38.7083**	**11.7020**	**105**	**-3.3080**	**0.0158**
**-4 vs. -1**	**-48.5833**	**11.7020**	**105**	**-4.1520**	**0.0009**
**-4 vs. +1**	**-45.3750**	**11.7020**	**105**	**-3.8780**	**0.0025**
-4 vs. +2	-10.5833	11.7020	105	-0.9040	0.9446
-3 vs. -2	-26.7083	11.7020	105	-2.2820	0.2106
**-3 vs. -1**	**-36.5833**	**11.7020**	**105**	**-3.1260**	**0.0270**
-3 vs. +1	-33.3750	11.7020	105	-2.8520	0.0571
-3 vs. +2	1.4167	11.7020	105	0.1210	1.0000
-2 vs. -1	-9.8750	11.7020	105	-0.8440	0.9585
-2 vs. +1	-6.6667	11.7020	105	-0.5700	0.9928
-2 vs. +2	28.1250	11.7020	105	2.4030	0.1645
-1 vs. +1	3.2083	11.7020	105	0.2740	0.9998
**-1 vs. +2**	**38.0000**	**11.7020**	**105**	**3.2470**	**0.0190**
**+1 vs. +2**	**34.7917**	**11.7020**	**105**	**2.9730**	**0.0414**

Bold text has been used to emphasize statistically significant differences. Weeks preceding parturition: -4 to -1; Weeks following parturition: +1 to +2.

## Discussion

The present investigation provides the first objective evidence of skin chroma and luminance variation noticeable during pregnancy in a New World primate. Our study reveals that throughout parturition, chromatic contrast between female sexual skin and the surroundings exhibits a “U” feature, only perceived by trichromatic females (Fig 3A), which may indicate the time of birth. The chromatic contrast disappears around parturition (two weeks before and one week after birth), dropping to imperceptible levels (Fig 2A), information that could warn related trichromatic females that new siblings are expected and that they should prepare themselves for alloparental care [47]. Still, since this variation in chromatic contrast over the weeks did not reach statistical significance, and since our limited sample size might have led to a large variance in our data, this finding should be interpreted with caution.

On the other hand, achromatic contrast in female sexual skin showed an inverted “U” feature (Fig 2B) that was supported by both our perceptual and statistical analyses. This luminance signal, which reaches very high values of achromatic contrast, can be well perceived, likewise, by all phenotypes (Fig 3B) found in this species, and might play a major role in reproductive signaling for conspecifics.

Since a post conception reproductive strategy of dominant females has been reported [48], based on committing infanticide when subordinate females give birth around dominant females’ parturition, we suggest that dominant females could use achromatic contrast as a birth timing cue of subordinate females. Alternatively, if preconception reproductive suppression is not imposed on subordinate females by dominants, but is instead self-imposed by most subordinates in response to the threat of infanticide [49], achromatic contrast of dominant females’ sexual skin could serve as an additional source of information for reproductive decision-making by subordinate females. Given that dichromatism is a condition found in all males and only a few females [50], we should also expect achromatic contrast to be very important to males, since they cannot exploit chromatic signals (Fig 3A, Table 1) and need to perceive the timing of birth in order to prepare themselves for paternal burdens, such as carrying offspring [25].

During pregnancy, female common marmosets show hormonal variation characterized by an acute decrease in estradiol and progesterone on the days preceding birth, as well as postpartum ovulation, which generally occurs within 10 to 20 days after delivery [51]. Interestingly, this hormonal profile coincides with the chromatic variation pattern (“U” feature) described in our data, suggesting a relationship between skin color and hormonal levels around parturition. On the other hand, this hormonal profile relates inversely to luminance variation.

If visual cues are adaptive for social communication, as we propose, then our results suggest that trichromatic females could have an advantage over dichromatic males and females (Fig 3A, Table 1). However, taking into account that levels of chromatic JND are much weaker than levels of achromatic JND (Figs 2 and 3), and that statistically significant variation throughout pregnancy has been found for achromatic, but not chromatic contrast (Table 2), it is likely that luminance variation alone plays a fundamental role in pregnancy detection, while chromatic variation should play little or no role in this signal. These conclusions are in accordance to previous suggestions that only variation in rhesus female facial luminance, not color, should be biologically relevant [41].

According to our achromatic and chromatic contrast results, dichromats with longer-wavelength pigments (430/562) and trichromats carrying widely separated pigments (430/543/562) could be more advantageous, although neither dichromatic phenotypes nor trichromatic phenotypes were found to differ perceptually within their own phenotype group. These advantages are consistent with what has previously been suggested for fruit detection [44, 52].

Earlier studies that have used subjective human perception when investigating animal socio-reproductive signals may have overestimated animal color variation [41]. Since only visual models and some behavioral experiments consider the view of the beholder [40, 41, 53], future studies on mate selection that combine hormonal and behavioral measurements with visual, olfactory and acoustic information should be encouraged. Future analyses that consider the relation of sexual hormones and skin coloration, in cycling and pregnant females, might bring even further contributions to the field.

## Supporting Information

S1 ARRIVE Checklist (PDF)

S1 Dataset Skin reflectance spectra from genitalia and thighs. Mean reflectance spectra (coloration) from the skin of the right and left sides of common marmoset female’s genitalia and thighs. Raw values given by Spectra Suite software (Ocean Optics). Weeks preceding (-4 to -1) and following (+1 to +2) parturition are indicated. wl = wavelenght. *Differences in wavelength scales reflect the use of different USB4000-UV-VIS spectrometers (Ocean Optics).
(XLSX)

S1 Fig Data collection procedure. A) Apparatus used to immobilize our subjects. Note that the animal has been habituated to the procedure and is calmly receiving its sugary reward. B) Body regions [right and left sides of female genitalia (red dots) and inner thighs (blue dots)] that have been subjected to reflectance spectra measurements.
(TIF)
