# Supplementary figures and images for: Parturition Signaling by Visual Cues in Female Marmosets (Callithrix jacchus)
Source: PLoS One. 2015 Jun 5;10(6):e0129319. doi: 10.1371/journal.pone.0129319 (PMC4457725; doi:10.1371/journal.pone.0129319)

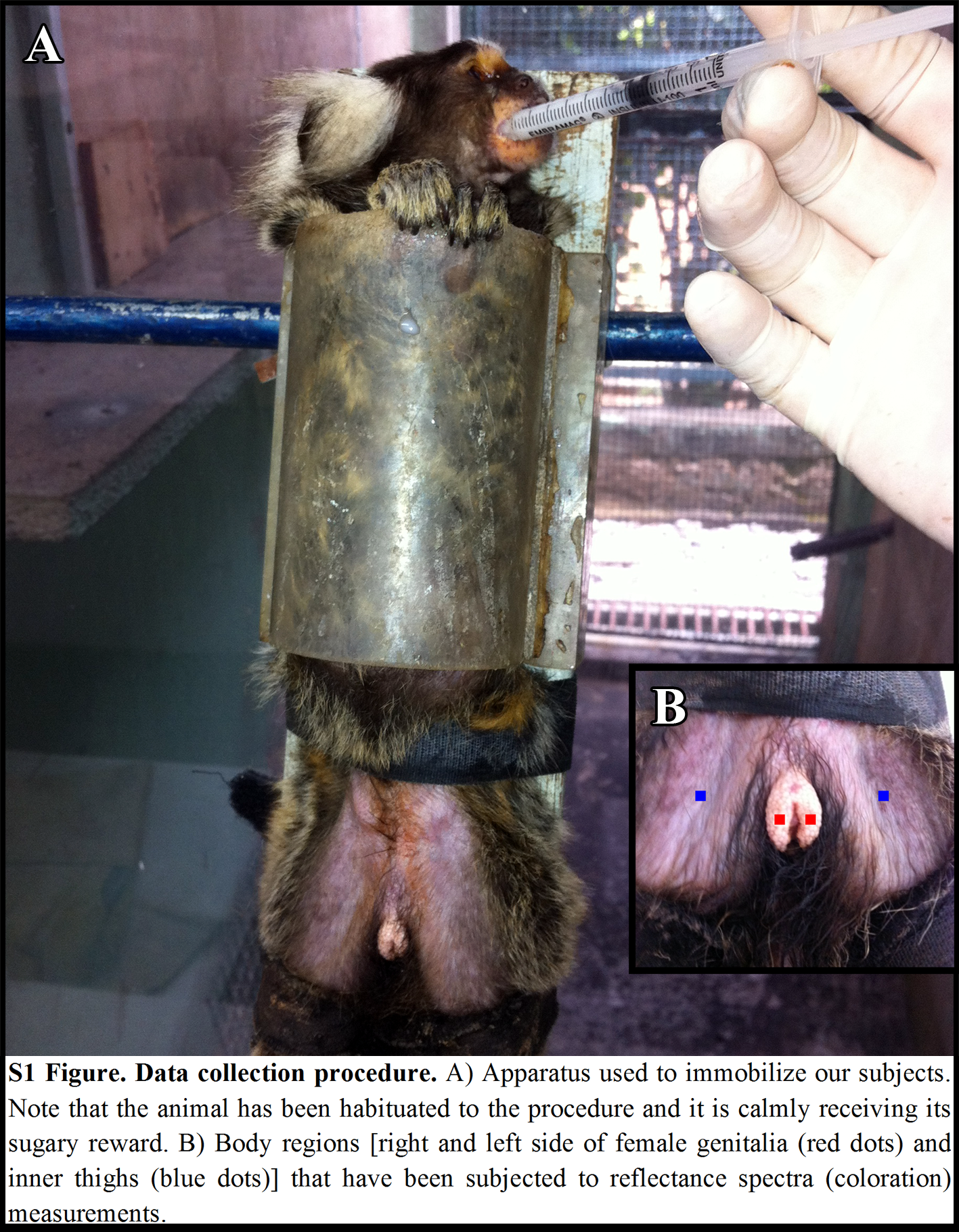

Supplement: S1 Fig — A) Apparatus used to immobilize our subjects. Note that the animal has been habituated to the procedure and is calmly receiving its sugary reward. B) Body regions [right and left sides of female genitalia (red dots) and inner thighs (blue dots)] that have been subjected to reflectance spectra measurements. (TIF) [file pone.0129319.s003.tif]
